# Generic Controlled-Release Budesonide in IgA Nephropathy – A Single Center Retrospective Study

**DOI:** 10.1016/j.ekir.2026.106368

**Published:** 2026-02-19

**Authors:** Soumita Bagchi, Dipankar Bhowmik, Sanjay Kumar Agarwal

**Affiliations:** 1 Department of Nephrology, All India Institute of Medical Sciences, New Delhi, India; 2 Department of Nephrology and Kidney Transplant Medicine, Marengo Asia Hospital, Gurugram, India

**Keywords:** generic controlled-release (CR) budesonide, IgA nephropathy, outcome

## Introduction

IgA nephropathy (IgAN) is the most common primary glomerular disease in adults worldwide. It has a smoldering trajectory with significant risk of progressive kidney failure. Recent breakthroughs have expanded the treatment armamentarium for IgAN.^1^ The therapeutic approach is potentially shifting from broad, nonspecific immunosuppression with steroids to targeted, disease mechanism-based treatments such as Tarpeyo (Nefecon), which has been recently approved for IgAN patients with progressive disease.^2^^,^^3^ Nefecon is a novel, targeted-release formulation of budesonide that acts locally in the ileum to modulate mucosal immunity. It inhibits the production of galactose-deficient IgA1 by gut-associated lymphoid tissue, while minimizing the adverse effects by limiting systemic steroid absorption. However, Nefecon remains unavailable or unaffordable, particularly in resource-limited regions such as South Asia, where IgAN is common and presents with an aggressive disease phenotype,^4^ thus limiting its relevance in routine clinical care. Avoiding systemic corticosteroid exposure is particularly important in these populations, as endemic levels of infectious diseases significantly increase the risk of treatment associated morbidity and mortality.

Repurposing controlled-release budesonide (CR-budesonide) formulations already approved and available for treatment of inflammatory bowel diseases may represent a pragmatic and affordable alternative in these regions. In this context, we discuss our experience with generic CR-budesonide formulations in Indian patients with difficult to treat IgAN.

## Results

We initially identified 65 patients who had received CR-budesonide during the assessed period (Supplementary Methods). We excluded 8 patients (5 had received other drugs concomitantly [hydroxychloroquine-3 and dapagliflozin-2], 2 were lost to follow-up immediately after starting treatment, and CR-budesonide was discontinued within 2 weeks in 1 patient because of COVID-19 infection). Analysis included all patients (*N* = 57) who had received budesonide for at least 1 month (Table 1).

**Table 1:** Baseline characteristics of patients at initiation of CR-budesonide therapy

Characteristics	*N* = 57^a^
Age (yrs)	35 (26, 42)
Males	40 (70.2)
Hypertension	42 (74%)
Time from kidney biopsy to treatment initiation (mos)	36 (11, 57)
Past history of immunosuppression use	8 (14)
Serum creatinine (mg/dl)	1.6 (1.2, 1.9)
eGFR (ml/min per 1.73 m^2^)	54 (41, 70)
Serum albumin(g/dl)	4.3 (4.1, 4.5)
Urine protein creatinine ratio (g/g)	1.9 (1.6, 2.5)
Hematuria	29 (51)
MEST-C lesions^b^	
M1	43 (75.4)
E1	09 (15.8)
S1	44 (77.2)
T1	18 (31.6)
T2	04 (70.1)
C1	06 (10.5)
Remission	
Complete remission	06 (10.5%)
Partial remission	19 (33.3%)

CR, controlled-release; MEST-C, mesangial hypercellularity score (M); endocapillary hypercellularity (E) segmental glomerulosclerosis (S); tubular atrophy/interstitial fibrosis (T) and cellular or fibrocellular crescents (C).

a Median (Q1, Q3); *n* (%).

b MEST-C score was reported for 53 patients.

**Figure 1:**
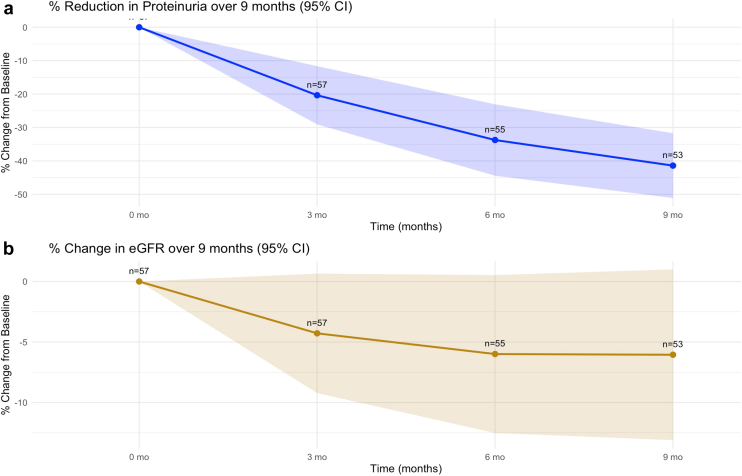
(a) Proteinuria over 9 months of CR-budesonide therapy (b) eGFR over 9 months of CR-budesonide therapy. CI, confidence interval; CR, controlled-release; eGFR, estimated glomerular filtration rate.

Median age was 35 years (inter quartile range [IQR] 26–42), and 40 (70.2 %) were males. Median estimated glomerular filtration rate (eGFR) was 54 ml/min per 1.73 m^2^ (IQR 41–70 ml/min per 1.73 m^2^) and proteinuria was 1.9 g/day (IQR 1.6–2.5 g/day). At the end of 9 months of treatment, 25 (43.8%) patients achieved remission of proteinuria (complete remission in 6 and partial remission in 19). There was a sustained reduction in proteinuria over 9 months (Figure 1a). eGFR remained stable during this period (Figure1b). CR-budesonide was associated with a −38.5% (SEM 5.5%) decrease in mean proteinuria from baseline (95% confidence interval −49.3% to −27.8%). eGFR remained relatively stable, with a mean change of −5.8% (SEM 3.5%; 95% confidence interval −12.7% to 1.1%).

Three patients had ≥ 40% decline in eGFR and none had progressed to end stage kidney disease . Seven patients required discontinuation of CR-budesonide and initiation of rescue therapy with alternative immunosuppression (all received prednisolone, and 4 also received mycophenolate mofetil). Mean percent reduction in proteinuria over 9 months was −45.0% (95% CI: −35.3% to –54.7%) in immunosuppression naïve patients (*n* = 47) and −26.0% (95% CI: −6.8% to −58.7%) in patients with previous history of immunosuppression (*n* = 10, *P* = 0.22).

Of the 25 patients who achieved remission, 14 (56%) experienced a relapse during median follow-up of 5.5 months (IQR 3–9.5) after withdrawal requiring reinitiation of CR-budesonide or alternate treatment. One patient developed mild edema and another had worsening hypertension, but neither required discontinuation of treatment. Although we did not assess for impaired glucose tolerance, none of the participants developed overt diabetes mellitus as defined by a fasting plasma glucose > 126 mg/dl during the follow-up period. Only 1 patient had a mild upper respiratory tract infection during treatment, and there was no history of any other serious infection including tuberculosis.

## Discussion

Our patient cohort had higher baseline proteinuria than the phase 3 Efficacy and Safety of Nefecon in Patients with Primary IgA Nephropathy (NefIgArd) trial population, where 97 patients on Nefecon 16 mg/day had a median urine protein creatinine ratio of 1.27 g/g and eGFR of 54.9 ml/min per 1.73 m^2^.^2^ Our patients showed a 38% reduction in proteinuria with no significant change in eGFR. In the NefIgArd trial, there was 31% reduction in proteinuria in intervention group, with 27% reduction along with a 3.87 ml/min per 1.73 m^2^ higher eGFR compared with placebo at 9 months. CR-budesonide (9 mg/day) also demonstrated sustained reduction in proteinuria and preservation of eGFR with minimal side effects in a previous study involving 32 patients.^5^ In an open-label randomized controlled trial in India,^6^ comparing CR-budesonide 18 mg/day plus standard of care (SOC), *N* = 27 with SOC (*N* = 26), CR-budesonide achieved superior reduction in proteinuria at 9 months (2441 ± 1048 mg → 813 ± 408 mg) compared with SOC (2386 ± 1057 mg → 1700 ± 672 mg; *P* < 0.001) and improvement in eGFR (80.0 ± 19.8 → 90.6 ± 11.8 ml/min per 1.73 m^2^) compared with a slight decline with SOC (82.2 ± 15.6 → 78.1 ± 12.5 ml/min per 1.73 m^2^). Adverse events were similar between groups, mainly mild gastrointestinal symptoms with 2 patients in the intervention arm developing prediabetic HbA1c and acne. Pharmacokinetic studies indicate that 3 mg of CR-budesonide is roughly equivalent to 10 mg of prednisolone, with a systemic bioavailability of 9 to 21%.^7^ It should be noted that CR-budesonide is a controlled-release formulation rather than a targeted-delivery steroid, which may influence systemic exposure. The recommended dose is 9 mg/day, and using higher doses, particularly for a prolonged period of 9 months or more and in repeated courses for relapses, should be approached with caution. Such dose escalation may increase systemic bioavailability and adverse effects, potentially undermining the drug’s key advantage of reduced systemic toxicity.

A key finding in our patient cohort was the absence of serious adverse events, especially infections, compared with conventional corticosteroids. Though systemic corticosteroids are widely used for IgAN in India, evidence on their efficacy and safety remains limited with no specific subgroup data available from the Therapeutic Evaluation of Steroids in IgA Nephropathy Global trial which included Indian patients.^8^ In a previous review of 94 patients(2012–2017) treated with prednisolone 1 mg/kg per day for 8 to 12 weeks followed by slow tapering at our center , 44.7% achieved remission followed by relapse or steroid dependence in 10 patients.^9^ However, in contrast to our experience with CR-budesonide, serious adverse effects occurred in 17% patients, predominantly infections, including 4 tuberculosis cases and 1 death from pulmonary nocardiosis.

This study had some limitations. This was a retrospective analysis from a single center with possibility of selection bias. There was no control group for comparison. Patients lacked contemporary biopsies at the time of treatment (median time from biopsy was 36 months; IQR 11–57). Notably, major IgAN trials, including NefIgArd, have also relied on historical biopsies ranging from a few years to nearly a decade old.^1^^,^^2^ Three different budesonide brands were used among our patients with the possibility of potential pharmacokinetic and pharmacodynamic variability among these formulations; however, such heterogeneity reflects routine clinical practice and is an inherent aspect of real-world data. Also as mentioned in the results, 7 patients required treatment with prednisolone alone or in combination with mycophenolate mofetil.

Despite these drawbacks, CR-budesonide seems to have substantial efficacy and was well tolerated. It has been successfully used in the treatment of other diseases, with a well-established safety profile. Its wide availability, affordability, and established safety make CR-budesonide a potentially effective, accessible, and safe alternative to conventional corticosteroids and Nefecon for the treatment of IgAN in low- and middle-income countries. This is particularly relevant as newer therapies often remain inaccessible to most patients in these resource limited regions. The use of such affordable alternatives may help improve outcomes and reduce health inequities in IgAN management. However, until further evidence supports higher dosing, the approved dose should be adhered to in order to minimize unnecessary systemic exposure. Prospective randomized controlled trials are needed to assess the long-term efficacy and safety of CR-budesonide formulations in IgAN patients.

## Disclosure

SB is a site investigator for trials in IgA nephropathy (Novartis, Vera Therapeutics and Fourrts). None of the other authors have any disclosures.

## Supplementary Material

Supplementary file (PDF) **Supplementary Methods.** STROBE Checklist.

## Footnotes

Supplementary file (PDF)

**Supplementary Methods.**
STROBE Checklist.
