# Supplementary material for: Generic Controlled-Release Budesonide in IgA Nephropathy – A Single Center Retrospective Study
Source: Kidney Int Rep. 2026 Feb 19;11(5):106368. doi: 10.1016/j.ekir.2026.106368 (PMC13022601; doi:10.1016/j.ekir.2026.106368)
Supplement: Supplementary file (PDF) — Supplementary Methods. STROBE Checklist. [file mmc1.pdf]

SUPPLEMENTARY METHOD

We conducted a retrospective, single-center study in the Department of Nephrology, All India Institute of Medical Sciences, New Delhi. Patients with biopsy-proven primary IgAN and persistent proteinuria ≥ 1 g/day despite optimized ACEi/ARB therapy and blood pressure ≤ 130/80 mmHg for at least 3 months, who had received CR-budesonide between 2018–2023, were identified from our clinical database. CR-budesonide therapy has been in use at our center since 2018, treatment being allocated by the physician based on clinical judgment and the patient's condition, without predefined eligibility criteria, reflecting the retrospective nature of the study.

The primary outcome was remission of proteinuria at 9 months. Partial remission was defined as proteinuria < 1 g/day with ≥ 50% reduction from baseline and stable eGFR (≤ 25% decline). Complete remission was defined as proteinuria < 0.5 g/day with stable eGFR. Relapse was defined as recurrence of proteinuria ≥ 1 g/day with or without eGFR decline after remission. Kidney disease progression was defined as > 40% sustained eGFR decline or kidney failure.

We also calculated the mean change with 95% confidence interval (CI) in proteinuria and eGFR over 9 months. Detailed methods are described in supplementary file.

Supplementary Method: In a retrospective study we reviewed the medical records of patients with biopsy proven primary IgAN who who had been treated with Controlled Release Budesonide(CR-budesonide) for persistent proteinuria ≥1g/day despite optimized angiotensin converting enzyme inhibitors (ACEi) /angiotensin receptor blockers (ARB) therapy and blood pressure control (≤130/80 mm of Hg) for at-least 3 months between 2018-2023. Patients with concomitant systemic diseases such as diabetes, chronic liver disease, an estimated glomerular filtration rate (eGFR) <30 mL/min/1.73 m² (calculated using the Chronic Kidney Disease Epidemiology Collaboration [CKD-EPI] formula), as well as a history of alternate immunosuppressive therapy in the preceding 6 months, were excluded from the study . Patients who had received CR-budesonide 9mg/day for at-least 1 months were included for analysis. The primary outcome assessed was remission of proteinuria at 9 months. Partial remission was defined as decrease in proteinuria to less than 1g/day with at-least 50% reduction from baseline and stable kidney function (≤25% reduction in eGFR) and complete remission as proteinuria <0.5 g/day. Progression of kidney disease was defined as more than 40% sustained decline in eGFR or end stage kidney disease(eGFR<10 ml/min/1.73m2 or requiring kidney replacement therapy). This analysis was part of a study approved by the institute ethics committee of All India Institute of Medical Sciences(IEC-240/16.05.2023) with waiver for informed consent.

Statistical Analysis: Continuous variables were summarized as mean ± standard deviation (SD) or median with interquartile range (IQR), as appropriate, and categorical variables were presented as counts and percentages. The primary analysis was conducted on an intention-to-treat basis, including all patients who received CR-budesonide for at least one month. The number of patients(%) who achieved partial and complete remission was determined. Changes in proteinuria and estimated glomerular filtration rate (eGFR) from baseline were analyzed using mean differences with standard error of the mean (SEM) and 95% confidence

intervals (CI). Percentage reduction in proteinuria over 9 months was compared between patients who had previous history of immunosuppression use followed by relapse and who were immunosuppression naive using mean ± 95% CI and Wilcoxon test. All statistical analyses were performed using R version 4.3.1 (R Studio). P value<0.05 was considered statistically significant

STROBE Statement—checklist of items that should be included in reports of observational studies

	Item No.	Recommendation	Page No.	Relevant text from manuscript
**Title and abstract**	1	(*a*) Indicate the study's design with a commonly used term in the title or the abstract	2	Methods:"We retrospectively analyzed…"
		(*b*) Provide in the abstract an informative and balanced summary of what was done and what was found	2	Methods and Results
**Introduction**				
Background/rationale	2	Explain the scientific background and rationale for the investigation being reported	3	Introduction
Objectives	3	State specific objectives, including any prespecified hypotheses	3	Introduction- "Repurposing generic controlled-release budesonide(CR-Budesonide) formulations already approved and available for treatment of inflammatory bowel diseases may represent a pragmatic and affordable alternative for the treatment of IgAN in these regions. In

				this context, we discuss our experience with generic CR-Budesonide formulations in Indian patients with difficult to treat IgAN”
**Methods**				
Study design	4	Present key elements of study design early in the paper	4, Supplementary file	Method and detailed methods in supplementary file
Setting	5	Describe the setting, locations, and relevant dates, including periods of recruitment, exposure, follow-up, and data collection	Supplementary file	Detailed Methods
Participants	6	(*a*) *Cohort study*—Give the eligibility criteria, and the sources and methods of selection of participants. Describe methods of follow-up *Case-control study*—Give the eligibility criteria, and the sources and methods of case ascertainment and control selection. Give the rationale for the choice of cases and controls *Cross-sectional study*—Give the eligibility criteria, and the sources and methods of selection of participants	Supplementary file	Inclusion and Exclusion criteria
		(*b*) *Cohort study*—For matched studies, give matching criteria and number of exposed and unexposed	NA	

		Case-control study—For matched studies, give matching criteria and the number of controls per case		
Variables	7	Clearly define all outcomes, exposures, predictors, potential confounders, and effect modifiers. Give diagnostic criteria, if applicable	Supplementary file	Detailed Methods
Data sources/ measurement	8*	For each variable of interest, give sources of data and details of methods of assessment (measurement). Describe comparability of assessment methods if there is more than one group	Supplementary file	Detailed Methods
Bias	9	Describe any efforts to address potential sources of bias	Supplementary file	Detailed Methods
Study size	10	Explain how the study size was arrived at	4	Methods

Continued on next page

Quantitative variables	11	Explain how quantitative variables were handled in the analyses. If applicable, describe which groupings were chosen and why	4	Method, Results
Statistical methods	12	(*a*) Describe all statistical methods, including those used to control for confounding	Supplementary File	Statistical Analysis
		(*b*) Describe any methods used to examine subgroups and interactions	NA	
		(*c*) Explain how missing data were addressed	NA	
		(*d*) *Cohort study*—If applicable, explain how loss to follow-up was addressed<br>*Case-control study*—If applicable, explain how matching of cases and controls was addressed<br>*Cross-sectional study*—If applicable, describe analytical methods taking account of sampling strategy	NA	No patient lost to follow up
		(*e*) Describe any sensitivity analyses	NA	Not done
**Results**				
Participants	13*	(a) Report numbers of individuals at each stage of study—eg numbers potentially eligible, examined for eligibility, confirmed eligible, included in the study, completing follow-up, and analysed	NA	All eligible patients were included
		(b) Give reasons for non-participation at each stage		
		(c) Consider use of a flow diagram		
Descriptive data	14*	(a) Give characteristics of study participants (eg demographic, clinical, social) and information on exposures and potential confounders	4, table 1	Results
		(b) Indicate number of participants with missing data for each variable of interest	NA	
		(c) *Cohort study*—Summarise follow-up time (eg, average and total amount)	4	Results
Outcome data	15*	*Cohort study*—Report numbers of outcome events or summary measures over time	*4*	*Results*
		Case-control study—Report numbers in each exposure		

		category, or summary measures of exposure		
		Cross-sectional study—Report numbers of outcome events or summary measures		
Main results	16	(*a*) Give unadjusted estimates and, if applicable, confounder-adjusted estimates and their precision (eg, 95% confidence interval). Make clear which confounders were adjusted for and why they were included	NA	
		(*b*) Report category boundaries when continuous variables were categorized	4, table 1	Results,(Median, IQR)
		(*c*) If relevant, consider translating estimates of relative risk into absolute risk for a meaningful time period	NA	

Continued on next page

Other analyses	17	Report other analyses done—eg analyses of subgroups and interactions, and sensitivity analyses	NA	
**Discussion**				
Key results	18	Summarise key results with reference to study objectives	5	Discussion
Limitations	19	Discuss limitations of the study, taking into account sources of potential bias or imprecision. Discuss both direction and magnitude of any potential bias	5	Discussion(Limitations)
Interpretation	20	Give a cautious overall interpretation of results considering objectives, limitations, multiplicity of analyses, results from similar studies, and other relevant evidence	5,6	Discussion
Generalisability	21	Discuss the generalisability (external validity) of the study results	6	Discussion
**Other information**				
Funding	22	Give the source of funding and the role of the funders for the present study and, if applicable, for the original study on which the present article is based	6	Funding

*Give information separately for cases and controls in case-control studies and, if applicable, for exposed and unexposed groups in cohort and cross-sectional studies.
